# Implications of cardiac markers in risk-stratification and management for COVID-19 patients

**DOI:** 10.1186/s13054-021-03555-z

**Published:** 2021-04-26

**Authors:** Pengping Li, Wei Wu, Tingting Zhang, Ziyu Wang, Jie Li, Mengyan Zhu, Yuan Liang, Wenhua You, Kening Li, Rong Ding, Bin Huang, Lingxiang Wu, Weiwei Duan, Yi Han, Xuesong Li, Xin Tang, Xin Wang, Han Shen, Qianghu Wang, Hong Yan, Xinyi Xia, Yong Ji, Hongshan Chen

**Affiliations:** 1 Department of Bioinformatics, Nanjing Medical University, Nanjing, China; 2 Department of Geriatrics, First Affiliated Hospital of Nanjing Medical University, Nanjing, China; 3 Key Laboratory of Cardiovascular and Cerebrovascular Medicine, School of Pharmacy, Nanjing Medical University, Nanjing, China; 4 Key Laboratory of Targeted Intervention of Cardiovascular Disease, Collaborative Innovation Center for Cardiovascular Disease Translational Medicine, Nanjing Medical University, Nanjing, China; 5 Faculty of Biology, Medicine and Health, The University of Manchester, Manchester, UK; 6 Department of Laboratory Medicine, Nanjing Drum Tower Hospital, The Affiliated Hospital of Nanjing University Medical School, Nanjing, China; 7 Jiangsu Key Lab of Cancer Biomarkers, Prevention and Treatment, Collaborative Innovation Center for Personalized Cancer Medicine, Nanjing Medical University, Nanjing, China; 8 Laboratory Medicine Center, the Second Affiliated Hospital, Nanjing Medical University, Nanjing, China; 9 COVID-19 Research Center, Institute of Laboratory Medicine, Jinling Hospital, Nanjing University School of Medicine, Nanjing, China; 10 Department of Laboratory Medicine & Blood Transfusion, Wuhan Huoshenshan Hospital, Wuhan, China; 11 Joint Expert Group for COVID-19, Wuhan Huoshenshan Hospital, Wuhan, China; 12 State Key Laboratory of Reproductive Medicine, Nanjing Medical University, Nanjing, China; 13 Department of Cardiothoracic Surgery, The Second Affiliated Hospital of Nanjing Medical University, Nanjing, China

**Keywords:** COVID-19, Cardiac markers, SARS-CoV-2 receptor

## Abstract

**Background:**

COVID-19 has resulted in high mortality worldwide. Information regarding cardiac markers for precise risk-stratification is limited. We aim to discover sensitive and reliable early-warning biomarkers for optimizing management and improving the prognosis of COVID-19 patients.

**Methods:**

A total of 2954 consecutive COVID-19 patients who were receiving treatment from the Wuhan Huoshenshan Hospital in China from February 4 to April 10 were included in this retrospective cohort. Serum levels of cardiac markers were collected after admission. Coronary artery disease diagnosis and survival status were recorded. Single-cell RNA-sequencing and bulk RNA-sequencing from different cohorts of non-COVID-19 were performed to analyze SARS-CoV-2 receptor expression.

**Results:**

Among 2954 COVID-19 patients in the analysis, the median age was 60 years (50–68 years), 1461 (49.5%) were female, and 1515 (51.3%) were severe/critical. Compared to mild/moderate (1439, 48.7%) patients, severe/critical patients showed significantly higher levels of cardiac markers within the first week after admission. In severe/critical COVID-19 patients, those with abnormal serum levels of BNP (42 [24.6%] vs 7 [1.1%]), hs-TNI (38 [48.1%] vs 6 [1.0%]), α- HBDH (55 [10.4%] vs 2 [0.2%]), CK-MB (45 [36.3%] vs 12 [0.9%]), and LDH (56 [12.5%] vs 1 [0.1%]) had a significantly higher mortality rate compared to patients with normal levels. The same trend was observed in the ICU admission rate. Severe/critical COVID-19 patients with pre-existing coronary artery disease (165/1,155 [10.9%]) had more cases of BNP (52 [46.5%] vs 119 [16.5%]), hs-TNI (24 [26.7%] vs 9.6 [%], α- HBDH (86 [55.5%] vs 443 [34.4%]), CK-MB (27 [17.4%] vs 97 [7.5%]), and LDH (65 [41.9%] vs 382 [29.7%]), when compared with those without coronary artery disease. There was enhanced SARS-CoV-2 receptor expression in coronary artery disease compared with healthy controls. From regression analysis, patients with five elevated cardiac markers were at a higher risk of death (hazards ratio 3.4 [95% CI 2.4–4.8]).

**Conclusions:**

COVID-19 patients with pre-existing coronary artery disease represented a higher abnormal percentage of cardiac markers, accompanied by high mortality and ICU admission rate. BNP together with hs-TNI, α- HBDH, CK-MB and LDH act as a prognostic biomarker in COVID-19 patients with or without pre-existing coronary artery disease.

**Supplementary Information:**

The online version contains supplementary material available at 10.1186/s13054-021-03555-z.

## Introduction

Coronavirus disease 2019 (COVID-19) is caused by the highly contagious severe acute respiratory syndrome coronavirus 2 (SARS-CoV-2) and has led to an ongoing global outbreak. Many affected patients develop interstitial pneumonitis and severe acute respiratory distress syndrome (ARDS), both associated with poor prognosis and high mortality [1, 2]. In addition to respiratory symptoms, patients also exhibit multi-organ dysfunction such as cellular immune deficiency, coagulation activation, and myocardial, hepatic, and kidney injury [3, 4].

With the rapid increase in confirmed cases, the cardiovascular manifestations induced by SARS-CoV-2 have generated considerable concern. A study of 138 hospitalized patients with COVID-19 showed that 7.2% had an acute myocardial injury [5]. Huang et al. reported that 12% of COVID-19 patients were diagnosed as having an acute myocardial injury [6]. COVID-19 patients with underlying coronary artery disease (CAD) who develop myocardial injury were found to have poorer in-hospital outcomes [7, 8]. However, sensitive and reliable markers for the early detection of myocardial damage and mortality risk assessment in patients with COVID-19 have not been well established. Moreover, to date, sensitive and reliable markers for the early detection of myocardial damage and mortality risk assessment in patients with COVID-19 have not been well established. Thus, a detailed analysis of clinical data is needed to identify early indicators of myocardial damage and mortality.

High-sensitivity troponin I (hs-TNI), α-hydroxybutyrate dehydrogenase (α-HBDH), creatine kinase-MB (CK-MB), and lactate dehydrogenase (LDH) are released into circulation when myocardial necrosis occurs and are, therefore, established as serum cardiac markers of myocardial injury [9]. In addition, serum levels of brain natriuretic peptide (BNP) can be an indicator of heart failure and utilized to differentiate non-cardiogenic from cardiogenic pulmonary edema [10, 11]. Several recent studies have investigated the association between hs-TNI and mortality in patients with COVID-19 [12, 13]. However, the association between the serum levels of cardiac markers and clinical outcomes in patients with and without pre-existing CAD has not been well established.

Previous studies have shown that angiotensin-converting enzyme 2 (ACE2) could be the receptor for SARS-CoV-2 [14]. It further confirmed that the SARS-CoV-2 could efficiently use ACE2 as a receptor for cellular entry, with an estimated 10- to 20-fold higher affinity to ACE2 than SARS-CoV [15, 16]. The receptor's expression and distribution decide the organ damage degree of virus infection, which has a significant implication for understanding its pathogenesis and designing therapeutic strategies[17]. Single-cell RNA sequencing (scRNA-seq) examines the gene expression information from individual cells with optimized next-generation sequencing technologies, providing a higher resolution of cellular differences and a better understanding of an individual cell's function. Some studies analyzed the mRNA expression profile of the SARS-CoV-2 receptor in different organs based on the public scRNA-seq database [17, 18].

In this single-center retrospective study from a cohort of 3,046 patients confirmed with COVID-19 at Wuhan Huoshenshan Hospital in China, the effectiveness of using cardiac markers including BNP, hs-TNI, α-HBDH, CK-MB, and LDH to predict mortality in patients with and without CAD upon admission was investigated. To characterize the expression patterns of SARS-CoV-2 receptors in the heart, scRNA-seq of non-COVID-19 cohorts was performed.

## Methods and materials

### Study design and participants

This single-center retrospective study included consecutive patients diagnosed with COVID-19 at Wuhan Huoshenshan Hospital in China, between February 4 and April 10, 2020. The Wuhan Huoshenshan Hospital was built in ten days due to the extent of the pandemic, which exceeded the existing health system capacity. Most of the patients admitted to the Huoshenshan Hospital were transferred from other hospitals. The study design was approved by the institutional ethics board. Written informed consent was waived due to the urgency of the COVID-19 pandemic.

**Fig. 1:**
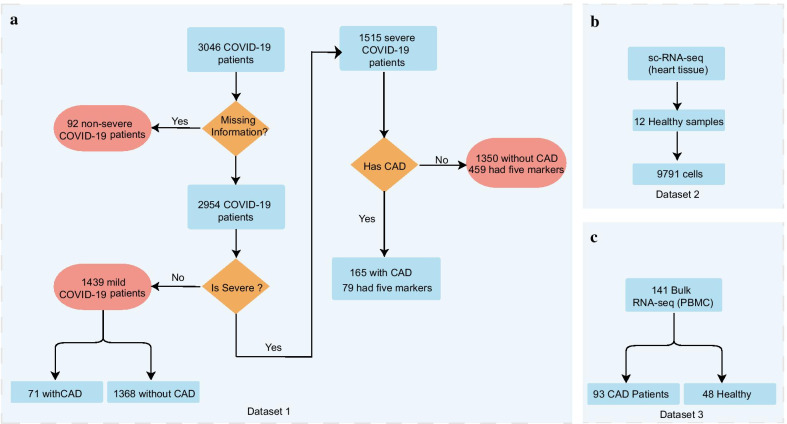
Flow chart of study design. **a** Flow chart of COVID-19 patients recruitment. **b** Single-cell RNA expression profile of SARS-CoV-2 receptors from 12 healthy human heart samples **(c)**. Bulk RNA expression profile of SARS-CoV-2 receptors from 93 CAD patients and 48 healthy control

The disease severity was determined according to the clinical classification criterion in the Diagnosis and Treatment Protocol for Novel Coronavirus Pneumonia released by the National Health Commission of China (7th edition, http://www.nhc.gov.cn/yzygj/s7653p/202003/46c9294a7dfe4cef80dc7f5912eb1989/files/ce3e6945832a438eaae415350a8ce964.pdf). Patients who met any of the following criteria during hospitalization were diagnosed as a severe case: (1) shortness of breath defined by respiration rate ≥ 30 breaths/min, (2) oxygen saturation ≤ 93 at rest, and (3) alveolar oxygen partial pressure/fraction of inspiration O_2_ (PaO_2_/FiO_2_) ≤ 300 mmHg (1 mmHg = 0.133 kPa). Patients whose pulmonary imaging showed significant progression of lesions > 50% within 24–48 h were also treated as severe cases. Patients who met any of the following conditions were diagnosed as critically severe: (1) respiratory failure requiring mechanical ventilation, (2) shock, and (3) organ failure needing intensive care unit (ICU) monitoring and treatment. In addition, the severe/critical cohort was categorized into two groups based on the presence or absence of pre-existing CAD (165/1515 [10.9%] and 1350/1515 [89.1%], respectively), according to clinical diagnosis and/or medical history on admission (Fig. 1a). Whether patients had pre-existing CAD or not was determined according to the description of CAD history in medical records on admission; New onset CAD was defined according to clinical diagnosis in medical records, such as “patient had undergone coronary angiography and had stenosis of non-major vessels, so the diagnosis of coronary heart disease was basically established”, or “Diagnostic basis of CAD: recurrent unstable angina attack, history of hypertension and diabetes for many years”. In 165 patients with CAD, 10 were new onset in which 8 were survivors; Suspected CAD in our study was based on electronic medical records with the descriptions as follows: “The recent occurrence of rapid atrial fibrillation which does not rule out the possibility of coronary artery disease”, or “a previous history of suspected coronary artery disease. Ten patients in CAD group and seven patients in non-CAD group had a history of congestive heart failure. Two patients in non-survivors and 14 patients in survivors with CAD had information of Ejection fraction (EF).

### Data collection

Clinical information was collected during hospitalization by attending physicians. The radiologic results including chest radiography or computed tomography (CT) were retrieved from the radiology information system documents. Patient data, including demographics, medical history, comorbidities, laboratory examinations, viral load such as ORF1ab and nucleocapsid (N), SARS-CoV-2-specific IgG and IgM antibodies, and outcomes were collected from electronic medical records and analyzed. The ORF1ab and nucleocapsid (N) genes were detected by performing real-time PCR assay and the number of cycles (CT value) is used to measure the viral load. A higher CT value indicates to a lower viral load. A CT value < 40 was defined as SARS-CoV-2 viral positive. Levels of IgM and IgG > 10 was defined as positive and ≤ 10 as negative [19].

### Cardiac biomarkers and myocardial damage

Cardiac biomarkers are substances released into the blood when the myocardial cell is damaged or stressed and can be useful in the early prediction or diagnosis of disease [20]. We used the common approach to handle missing data which is to omit those cases with the missing data and analyzed the remaining data [21]. For each marker, patients with missing value were removed. We dichotomizing the continuous data of the specific cardiac biomarker for each patient into categories, e.g., normal or abnormal, by using the approaches to modelling continuous variable [22–24]. Abnormal cardiac biomarker results were defined as serum levels above the upper reference limit.

### Transcriptional profiles

To characterize the expression patterns of SARS-CoV-2 receptors in the heart, scRNA-seq of 9791 cells isolated from 12 normal human hearts of non-COVID-19 cohorts was performed (Fig. 1b). The scRNA-seq datawere obtained from the Gene Expression Omnibus (GEO) database with the accession number GSE109816 [25] and processed using the *Seurat* toolkit [26].

Furthermore, to compare the gene expression difference of the SARS-CoV-2 receptor between the CAD patients and the non-CAD patient, we analyzed the bulk RNA expression profile of SARS-CoV-2 receptors in the peripheral blood mononuclear cells (PBMC). The expression profile of peripheral blood from 93 CAD patients and 48 healthy individuals of non-COVID-19 cohorts (Fig. 1c) which were approved by separate Institutional Review Board (IRB) was downloaded from the GEO database with the accession number GSE113079 [27] and was analyzed using the Agilent-067406 Human Microarray V4.0 platform (Aglient, Santa Clara, USA).

### Statistical analysis

Continuous and categorical variables are presented as median (interquartile range [IQR]) and number (%), respectively. Comparisons between groups were performed using the Mann–Whitney *U* test, χ^2^ test, or Fisher’s exact test where appropriate. Pearson’s correlations were employed to study the association between laboratory parameters. All statistical analyses were conducted using R software. Multivariate Cox regressions, log-rank tests, and Kaplan–Meier curves to plot the cumulative rates of death were implemented using the *Survival* package in R software*.* The receiver operating characteristic (ROC) curve to assess the overall accuracy of a prognostic marker was applied by using the R package ROCR. A *P*-value < 0.05 was used to indicate statistical significance.

## Results

### Baseline clinical characteristics

Of the initial 3046 patients with COVID-19 enrolled in our study, 48 patients with no record of survival status, 29 patients without classification of disease severity, and 15 patients with suspected CAD but with no diagnosis were excluded. In the remaining cohort of 2954 patients, 1439 patients were mild/moderate cases and 1515 were severe/critical cases. Among 1439 mild/moderate cases, 71 had CAD. 538 severe/critical cases had complete biomarker data during hospitalization, of which 79 had CAD (Fig. 1a).

Among the final cohort of 2954 patients, the median age was 60 years (range 50–68 years), 1461 (49.5%) were female, and 1515 (51.3%) were severe/critical cases. The median hospital stay for severe/critical (S) patients was significantly longer than that for mild/moderate (M) cases. Compared with mild/moderate cases, severe/critical patients were more likely to experience chest tightness. Comorbidities were more prevalent among severe/critical patients compared to mild/moderate cases, including hypertension, diabetes, cardiovascular disease, cerebrovascular disease, cancer, and chronic obstructive pulmonary disease (Table 1).

**Table 1:** Demographics and clinical characteristics of patients with COVID-19

	Total (N = 2954)	Mild/Moderate (N = 1439)	Severe/Critical (N = 1515)	P value
*Characteristics*				
Age (yr.)—median (Interquartile range [IQR])	60 (50–68)	57(45–65)	63 (54–71)	< 0.001
Sex– no. (%)	–	–	–	1
Male	1,493 (50.5%)	728 (50.6%)	765 (50.5%)	
Female	1,461 (49.5%)	711 (49.4%)	750 (49.5%)	
Initial temperature (℃) – median (IQR)	–	–	–	0.002
≤ 37.3– no. (%)	2,833 (95.9%)	1,398 (97%)	1,435 (94.7%)	–
37.3–38– no. (%)	85 (2.9%)	25 (2%)	60 (4%)	–
38 -39– no. (%)	35 (1.17%)	16 (1%)	19(1.2%)	–
> 39– no. (%)	1 (0.03%)	0 (0%)	1 (0.1%)	–
Comorbidities—no. (%)				
Hypertension	889 (30%)	348 (24%)	541 (36%)	< 0.001
Diabetes	401 (14%)	159 (11%)	242 (16%)	< 0.001
Cardiovascular disease	330 (11%)	104 (7%)	226 (15%)	< 0.001
Cerebrovascular disease	118 (4%)	34 (2%)	84 (6%)	< 0.001
Cancer	74 (3%)	19 (1%)	55 (4%)	< 0.001
Chronic obstructive pulmonary disease	139 (5%)	40 (3%)	99 (7%)	< 0.001
Chronic renal disease	47 (2%)	14 (1%)	33 (2%)	0.012
Chronic liver disease	76 (3%)	36 (3%)	40 (3%)	0.817
Immunodeficiency	9 (0%)	4 (0%)	5 (0%)	1
Respiratory rate—median (IQR)	–	–	–	< 0.001
> 24 breaths per min– no. (%)	132 (4%)	14 (1%)	118 (8%)	–
≤ 24 breaths per min–no. (%)	2,820 (95%)	1,425 (99%)	1,395 (92%)	–
Hospital stays—median (IQR)	13 (8–19)	12 (8–17)	14 (8–22)	< 0.001
Death—no. (%)	59(2%)	0 (0%)	59(4%)	< 0.001
Clinical symptoms—no. (%)				
Fever	2,149 (73%)	1,032 (72%)	1,117 (74%)	0.231
Cough	2,062 (70%)	992 (69%)	1,070 (71%)	0.336
Shortness of breath	1302 (44%)	608 (42%)	694 (46%)	0.05
Chest tightness	1,137 (38%)	517 (36%)	620 (41%)	0.006
Fatigue	669 (23%)	279 (19%)	390 (26%)	< 0.001
Muscle soreness	118 (4%)	29 (2%)	89 (6%)	< 0.001
Headache	17 (1%)	13 (1%)	4 (0%)	0.027
Dizziness	26 (1%)	14 (1%)	12 (1%)	0.695
Nausea	22 (1%)	12 (1%)	10 (1%)	0.671
Vomiting	22 (1%)	11 (1%)	11 (1%)	1
Diarrhea	64 (2%)	34 (2%)	30 (2%)	0.528
Radiological findings (N = 2567)—no. (%)				
Blurred edges	304 (12%)	118 (9.6%)	186 (14%)	< 0.001
Lymph node enlargement	30 (1.2%)	18 (1.5%)	12 (0.9%)	0.202
Ground glass opacity	2324 (91%)	1109 (90%)	1215 (91%)	0.304
Cystic change	209 (8.2%)	81 (6.6%)	128 (9.6%)	0.006
Airway obstruction	4 (0.16%)	2 (0.16%)	2 (0.15%)	1
Consolidation	332 (13%)	115 (9.4%)	217 (16%)	< 0.001
Fine reticular opacity	56 (2.2%)	17 (1.4%)	39 (2.9%)	0.01
Lung texture increase	1529 (60%)	734 (60%)	795 (60%)	0.968
Intralobular septal thickening	14 (0.55%)	4 (0.33%)	10 (0.75%)	0.183
Pleural thickening	221 (8.6%)	72 (5.9%)	149 (11%)	< 0.001
Pleural effusion	205 (8%)	34 (2.8%)	171 (13%)	< 0.001
Bronchiectasis	53 (2.1%)	17 (1.4%)	36 (2.7%)	0.025
Viral load of throat swabs (N = 1476)—median (IQR)				
ORF1ab	37.7 (34.51–40.08)	37.85 (34.85–40.05)	37.62 (34.07–40.15)	0.258
N	36.33 (33.77–37.82)	36.4 (34.08–37.77)	36.28 (33.5–37.91)	0.762
Anti-SARS-Cov-2 IgG and IgM level—median (IQR)				
IgG level	139.49 (73.93–181.24)	141.33 (74.82–180.45)	137.64 (73.88–181.45)	0.864
IgM level	26.88 (8.38–66.74)	26.46 (8.13–62.5)	27.32 (8.52–68.62)	0.337

**Fig. 2:**
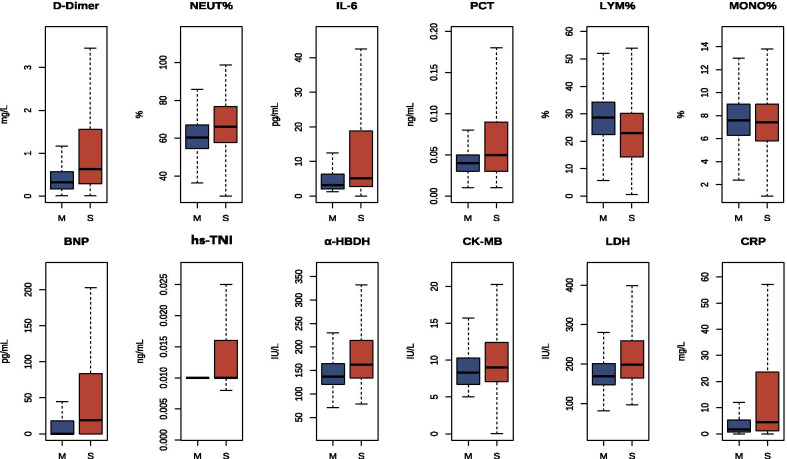
Serum cardiac markers drastically elevated in severe/critical patients during the first week. Levels of 12 multiple organ dysfunction indicators in mild/moderate (M) and severe/critical (S) groups during the first week of hospitalization. The difference of each indicator between the two groups is significant (two-sided Wilcoxon test, *P* < 0.001)

In terms of radiological and laboratory findings, severe/critical (S) patients had more incidences of blurred edges and consolidation (Table 1) and significantly higher levels of C-reactive protein (CRP), D-dimer, interleukin-6 (IL-6), procalcitonin (PCT), and higher percentages of neutrophils (NEUT%), lymphocytes (LYM%), and monocytes (MONO%) within the first week of admission (Fig. 2). Serum cardiac markers, namely; BNP, hs-TNI, α-HBDH, CK-MB, and LDH, were also drastically elevated in severe/critical patients during the first week (Fig. 2). In general, the results showed more pronounced activation of pathophysiological pathways in more severe cases of COVID-19.

### Cardiac markers and clinical outcomes

To evaluate the relationship between the degree of cardiac abnormality and disease outcome in patients with COVID-19, serum cardiac markers during hospitalization were measured. Due to the 0% mortality and favorable prognosis of mild/moderate patients, we focused on the 1,515 patients with severe/critical COVID-19 in the follow-up period.

Five cardiac biomarkers, namely, BNP, hs-TNI, α-HBDH, CK-MB, and LDH, were collected to evaluate heart function. For the detection of BNP, 1456 samples from 835 patients were tested during hospitalization. Each patient was tested 1 to 19 times, and 281 (33.6%) were tested more than once. The median sampling interval was 4 days among patients who were tested more than once. For the detection of α-HBDH and LDH, 3900 samples from 1443 patients were tested. Each patient was tested 1 to 27 times, and 835 (57.9%) were tested more than once. The median sampling interval was 4 days among patients who were tested more than once. For the detection of total CK-MB, 3885 samples from 1442 patients were tested. Each patient was tested 1 to 27 times, and 834 (57.8%) were tested more than once. The median sampling interval was 4 days among patients who were tested more than once. For the detection of total hs-Tnl, 1126 samples from 660 patients were tested. Each patient was tested 1 to 18 times, and 209 (31.6%) were tested more than once. The median sampling interval was 4 days among patients who were tested more than once. A total of 538 critical/severe patients had all five cardiac markers measured during hospitalization.

**Fig. 3:**
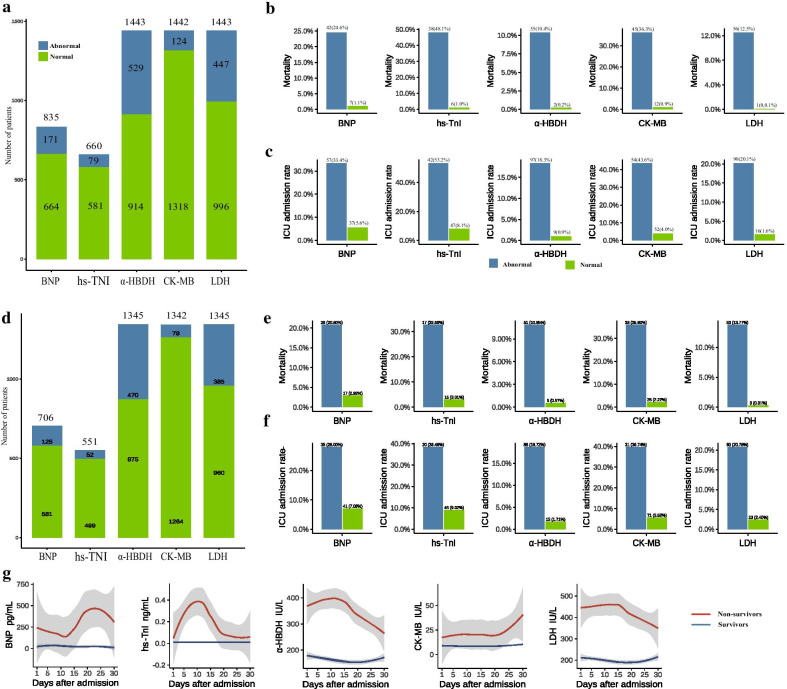
Severe/critical COVID-19 patients with evidence of abnormal cardiac markers have poor clinical outcomes. **a** Number of patients with abnormal and normal levels of BNP, hs-TNI, α-HBDH, CK-MB, and LDH during hospitalization. The mortality **(b)** and ICU admission rate **(c)** of severe/critical COVID-19 patients with abnormal serum levels of BNP, hs-TNI, α-HBDH, CK-MB, and LDH during hospitalization. **d** Number of patients with abnormal and normal levels of BNP, hs-TNI, α-HBDH, CK-MB, and LDH within the first week after admission. The mortality **(e)** and ICU admission rate **(f)** of severe/critical COVID-19 patients with abnormal serum levels of BNP, hs-TNI, α-HBDH, CK-MB, and LDH within the first week after admission. **g** Serum levels of BNP, hs-TNI, α-HBDH, CK-MB, and LDH during hospitalization for non-survivors and survivors. Shaded regions represent a 95% confidence interval

In total, 171 (20.5%), 79 (12.0%), 529 (36.7%), 124 (8.6%), and 447 (3.1%) patients showed abnormal serum levels of BNP, hs-TNI, α-HBDH, CK-MB, and LDH, respectively (Fig. 3a). Patients with an elevated level of a cardiac marker during hospitalization showed a significantly higher mortality than those with normal serum levels (Fig. 3b). The same trend was observed in the ICU admission rate (Fig. 3c).

When considering detection data within the first week after admission, BNP, hs-TNI, α-HBDH, CK-MB, and LDH were detected in 706 patients, 551 patients, 1345 patients, 1342 patients, and 1345 patients, respectively. 125 (17.8%), 52 (9.4%), 470 (35.0%), 78 (5.8%), and 385 (28.7%) patients showed abnormal serum levels of BNP, hs-TNI, α-HBDH, CK-MB, and LDH (Fig. 3d). Patients with an elevated level of cardiac markers within the first week after admission also showed a significantly higher mortality (Fig. 3e) and ICU admission rate than those with normal serum levels (Fig. 3f). Figure 3g shows that the serum levels of BNP, hs-TNI, α-HBDH, CK-MB, and LDH were significantly higher during hospitalization in non-survivors than in survivors. The results represented patients having abnormal level of cardiac markers within the first week after admission and during hospitalization may predict deterioration or progression.

### Expression of SARS-CoV-2 receptors in heart tissue of non-COVID-19 cohorts

**Fig. 4:**
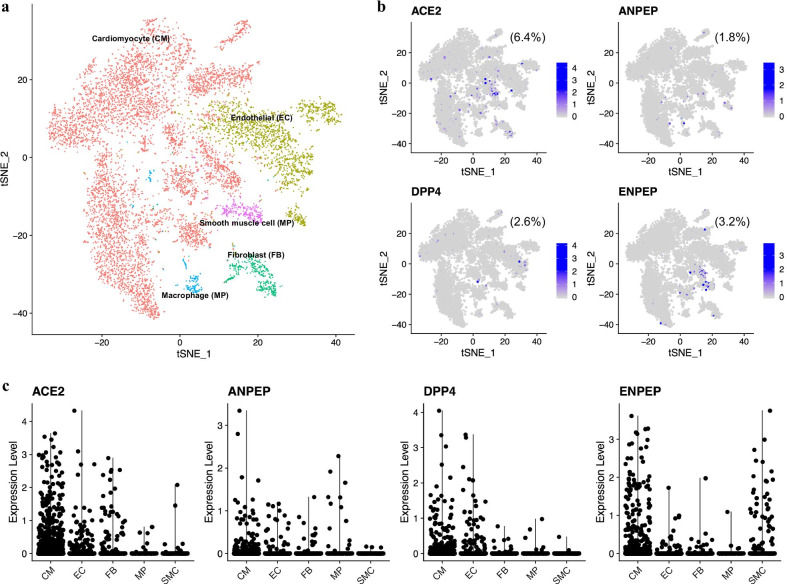
scRNA-seq analysis of SARS-CoV-2 receptors in heart specific cell populations. **a** The t-distributed Stochastic Neighbor Embedding (t-SNE) diagram shows the main cell types in healthy heart tissue. Each dot represents a cell, and each color represents a cell type. **b** Feature maps and **c** violin diagrams show SARS-CoV-2 receptors are enriched in specific cell populations in healthy heart tissues

The scRNA-seq data of normal human heart tissue were analyzed and five cell types, namely, cardiomyocyte (CM), endothelial (EC), fibroblast (FB), macrophage (MP), and smooth muscle (SMC) cells, were identified (Fig. 4a). SARS-CoV-2 receptors ACE2, ANPEP, DPP4, and ENPEP were enriched in specific cell populations. ACE2 was mainly expressed in CM, EC, and FB cell types. ANPEP was enriched in CM, DPP4 was mainly expressed by CM and EC, and ENPEP was primarily expressed in CM and SMC (Fig. 4b, c).

### Cardiac markers and mortality rate in patients with CAD or not

**Fig. 5:**
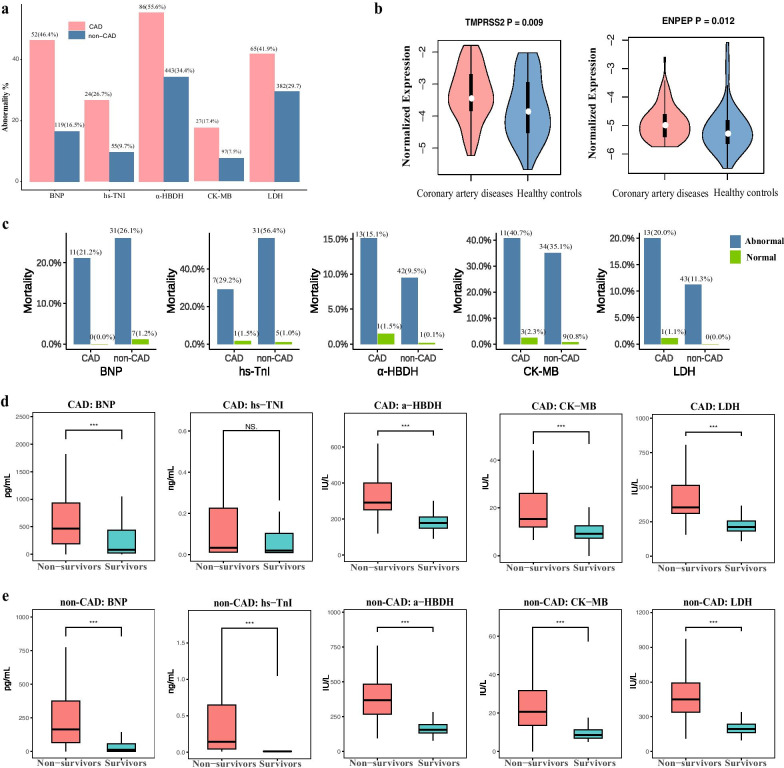
Severe/critical COVID-19 patients with abnormal cardiac markers exhibited higher mortality. **a** Percent of abnormal cardiac markers in patients with and without pre-existing CAD. **b** Expression of SARS-CoV-2 receptors, TMPRSS2 and ENPEP, in coronary artery disease and healthy controls. **c** The mortality rate of COVID-19 patients with cardiac markers abnormality. **d**–**e** Median levels of cardiac markers in survivors and non-survivors during hospitalization. ***P < 0.001; NS: not significant

The 1,515 severe/critical patients were further categorized into groups according to the presence of pre-existing CAD (*n* = 165) or absence (*n* = 1350). Compared with patients without CAD, patients with pre-existing CAD had a higher percent of elevated BNP (52 [46.4%] vs 119 [16.5%]), hs-TNI (24 [26.7%] vs 55 [9.7%], α- HBDH (86 [55.6%] vs 443 [34.4%]), CK-MB (27 [17.4%] vs 97 [7.5%]), and LDH (65 [41.9%] vs 382 [29.7%]) during hospitalization (*P* < 0.01 for all results, (Fig. 5a). Compared to patients with normal levels of cardiac markers, those with abnormal levels of BNP, hs-TNI, α-HBDH, CK-MB, and LDH during hospitalization exhibited significantly higher mortality in both CAD and non-CAD groups (*P* < 0.001 for all results; Fig. 5c). The same trend was observed for the ICU admission rate (Additional file 2: Figure S1). The serum markers were then compared between non-survivors and survivors. The results showed that BNP, α-HBDH, CK-MB, and LDH were significantly higher in non-survivors than in survivors for patients with pre-existing CAD (Fig. 5d). In patients without pre-existing CAD, all markers were significantly higher in non-survivors than in survivors during hospitalization (Fig. 5e).

### SARS-CoV-2 receptors in CAD and healthy controls of non-COVID-19 cohorts

To explore the underlying pathophysiological mechanism for the elevated levels of cardiac markers in COVID-19 patients with pre-existing CAD, RNA-seq data from 93 patients with CAD and 48 healthy people were analyzed and compared. The results showed that compared with healthy controls, SARS-CoV-2 receptors, including Transmembrane Serine Protease 2 (TMPRSS2, *P* = 0.009) and Glutamyl Aminopeptidase (ENPEP, *P* = 0.012), were significantly upregulated in CAD (Fig. 5b).

### Cardiac markers in non-survivors with CAD or not

Notably, the median value of BNP was significantly higher in non-survivors than in survivors for those with pre-existing CAD (911.3 pg/mL vs. 57.9 pg/mL) and those without (121 pg/mL vs. 0.01 pg/mL) on the first day after admission. The median levels of hs-TNI, α-HBDH, and LDH were significantly higher in non-survivors than in survivors in patients without pre-existing CAD; however, a significant difference was not observed in patients with pre-existing CAD (Additional file 3: Figure S2).

**Fig. 6:**
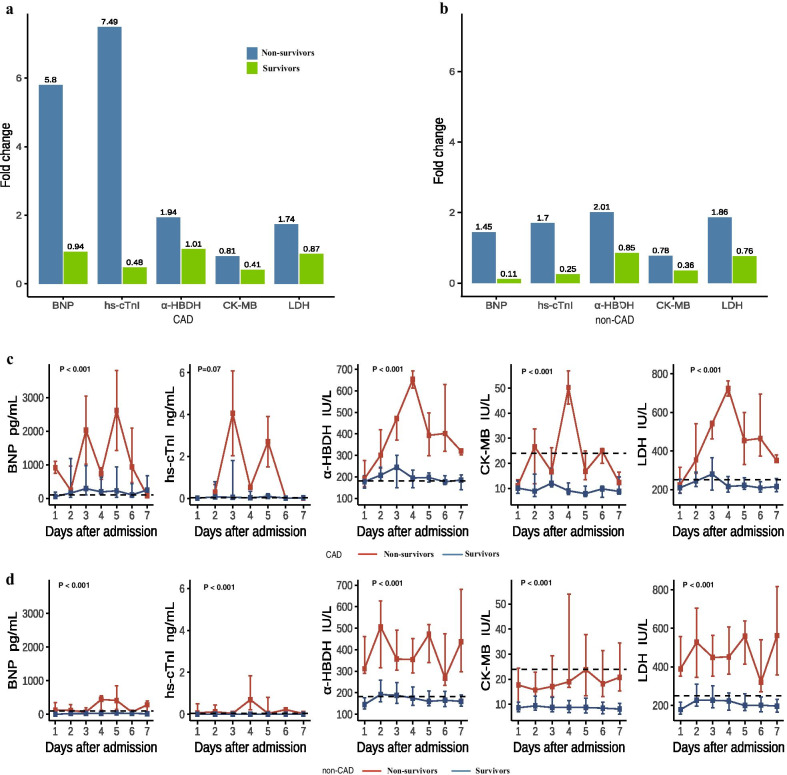
Risk-Stratification Biomarker for COVID-19 patients. **a–b** Fold change of cardiac markers relative to the upper reference within one week after admission. **c–d** The fluctuation of serum levels of cardiac markers within one week after admission. CAD: COVID-19 patients with pre-existing CAD; non-CAD: COVID-19 patients without pre-existing CAD

In non-survivors with pre-existing CAD, the median levels of BNP and hs-TNI within the first week showed a higher fold change (BNP: 5.8; hs-TNI: 7.5; α-HBDH: 1.9; CK-MB: 0.81; LDH: 1.7) from the upper reference limit of each marker (Fig. 6a). In non-survivors without pre-existing CAD, the fold change for BNP, hs-TNI, α-HBDH:1.9, CK-MB, LDH was 1.4, 1.7, 2, 0.78, and 1.9, respectively (Fig. 6b). The serum level differences for the five markers within a week after admission in patients with or without pre-existing CAD showed that BNP, α-HBDH, and LDH values were significantly higher in non-survivors than in survivors regardless of pre-existing CAD (Fig. 6c, d). Levels of hs-TNI were significantly higher only in non-survivors than in survivors for patients without pre-existing CAD. Although CK-MB were significantly different between non-survivors and survivors, most data for non-survivors were within normal levels (Fig. 6c, d). The change of five markers levels was the same as those mentioned above during hospitalization (Additional file 4: Figure S3).

### Elevated cardiac markers and increased mortality

The mortality between patients with high and normal cardiac markers within the first week after admission that were not in the ICU were analyzed. When we retrieved patients’ electronic medical records, we found 4 patients with elevated five markers were not admitted into ICU and 3 patient died. Conversely, 217 patients with normal level of five cardiac markers were not admitted into ICU and 0 died. The results demonstrated the mortality of patients with elevated five cardiac markers not admitted to ICU was significantly higher than those with normal cardiac markers not admitted to ICU (Fisher exact test, *P* < 0.0001). Additionally, 90 patients with elevated BNP, 32 patients with elevated hs-TNI, 382 patients with elevated α-HBDH, 47 patients with elevated CK-MB and 305 patients with elevated LDH within the first week were not admitted to ICU, respectively (Additional file 1: Table S1, Fig. 3f).

**Fig. 7:**
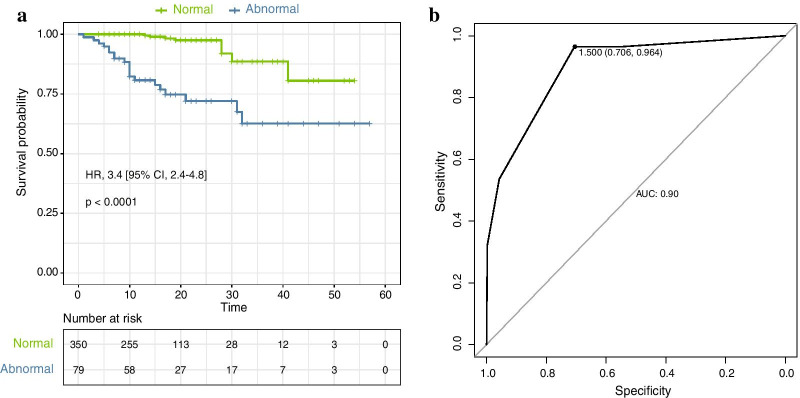
Abnormal levels of five cardiac makers is correlated with increased mortality of COVID-19 patients. **a** Kaplan–Meier estimates for severe/critical patients by levels of BNP, hs-TNI, α-HBDH, CK-MB, and LDH within the first week after admission in multivariate Cox regression analysis. Log-rank test shows statistical significance. The table below shows the number of people still alive at different time points. **b** ROC curve of five cardiac markers to predict survivors and non-survivors

In order to evaluate the risk of elevated levels of cardiac markers in COVID-19 patients as early as possible, we established a prognosis prediction model based on levels of BNP, hs-TNI, α-HBDH, CK-MB, and LDH within the first week after admission. Firstly, univariate Cox regression analysis was conducted by applying a mixed-effect Cox model adjusted for age, sex, comorbidities for each cardiac marker. Patients with missing values for each marker were removed. We identified five markers were independent predictor of mortality (*P* < 0.001, Additional file 5: Figs. S4 and S5). Secondly, to predict the risk of mortality more accurately, five markers were entered into a multivariate Cox regression analysis. Samples with missing values in any marker were removed and 429 patients have five cardiac markers detections were included in the subsequent analysis. We divided 429 patients into cardiac markers abnormal (*n* = 79) and normal (*n* = 350) groups based on the upper reference limit. As a result, the five cardiac markers abnormal group demonstrated a significantly higher risk of mortality with hazards ratio 3.4 [95% CI, 2.4–4.8] (Fig. 7a). Five cardiac markers levels classified survivors and non-survivors with an area under the curve (AUC) of 0.90 with the threshold value based on the ROC curve of 1.5, a sensitivity of 70.6%, and a specificity of 96.4% (Fig. 7b).

CRP, D-dimer, and IL-6 are commonly elevated and LYM% diminished in patients with COVID-19 and correlate with disease severity in previous studies. We analyzed the correlation between five cardiac barkers and CRP, D-dimer, IL-6, and LYM%. The results showed BNP, hs-TNI, α-HBDH, CK-MB, and LDH were highly positive correlation with CRP, IL-6, and D-dimer (Additional file 7: Fig. S6). By contrast, five markers were significantly negatively correlated with LYM% (Fig. S6).

## Discussion

Our study provides detailed information about the association between cardiac markers and clinical outcomes of COVID-19 patients with or without pre-existing CAD. Patients with abnormal serum levels of cardiac markers, namely, BNP, hs-TNI, α-HBDH, CK-MB, and LDH, had a significantly higher mortality rate than patients with normal serum marker levels. In COVID-19 non-survivors with or without pre-existing CAD, the BNP level measured within one week after admission showed a 5.8-fold and 1.4-fold significant increase from the upper reference limit, respectively. Non-survivors with CAD had no hs-TNI detection on admission, this may be the reason hs-TNI in this cohort showed a delayed abnormal course compared to other biomarkers. Our results demonstrated that BNP together with hs-TNI, CK-MB, and LDH within the first week after admission could act as risk factors for in-hospital mortality.

SARS-CoV-2 uses the ACE2 receptor to facilitate viral entry into target cells, causing multiorgan dysfunction [4]. It has been reported that the mechanism of acute myocardial injury caused by SARS-CoV-2 infection might be related to ACE2 [28]. The scRNA-seq analysis in the present study showed that ACE2 was mainly expressed in CM, EC, and FB cell types, suggesting that SARS-CoV-2 receptor-related signaling pathways in myocardial injury may be mainly related to these cell populations. Whether someone has CAD or not, once the patient is infected with the virus, the heart may be severely attacked.

Hoffman et al. [29] recently demonstrated that the initial spike in protein priming by TMPRSS2 is essential for the entry and viral spread of SARS-CoV-2 through interaction with the ACE2 receptor [30]. Compared with COVID-19 patients without CAD, patients with pre-existing CAD had a higher percent of elevated BNP, hs-TNI, α- HBDH, CK-MB, and LDH. There is no difference in mortality between COVID-19 patients with CAD and non-CAD when BNP showed abnormal. However, the difference was observed when they represented elevated α- HBDH, CK-MB, and LDH. A significantly higher expression of TMPRSS2 in CAD patients compared with healthy controls suggested that CAD patient may have a higher risk of SARS-CoV-2 infection than health control. It implies that upregulated TMPRSS2 may be the reason that patients with CAD represented a higher percentage of individuals with abnormal levels of cardiac markers after SARS-CoV-2 infection. The serine protease inhibitor camostat mesylate, approved in Japan to treat diseases, has been shown to block TMPRSS2 activity and is thus an interesting candidate to treat COVID-19 patients with pre-existing CAD [31, 32].

Previous studies have investigated the association between myocardial injury markers and prognosis [33, 34]. However, evidence on markers that are appropriate for monitoring myocardial injury in COVID-19 patients timely is lacking. Because of limited space in ICUs, the triage rules for access to intensive care are becoming tougher and tougher.

The results in this study showed that BNP together with hs-TNI, α-HBDH, CK-MB, and LDH within the first week after admission could be used as biomarker for prognosis in COVID-19 patients. These cardiac biomarkers were strongly correlated with inflammatory markers, indicating that cardiac biomarkers may track with overall severity of illness and multisystem organ dysfunction. The first week of admission could be a window for observation and early intervention, i.e. that five markers will precede clinical deterioration or escalation of care.

The survival rate of mild/moderate patients is 100% in our cohort which is the same as previous studies, whose data collected from Wuhan Huoshenshan Hospital in Wuhan, China [35]. Huoshenshan Hospital is an emergency specialty field in response to COVID-19 pandemic in China that could make quick and informed decisions for patients. Mild/moderate patients could receive adequate care and timely treatment after admission. Therefore, it is maybe a significant reason for none of the mild/moderate COVID-19 patients progressed to death.

Some limitations exist in the present study. First, there was some indication bias to exclude cases with missing biomarker data from regression analyses, as sicker cases were having more intensive monitoring of these parameters and so they were more likely to be measured, in addition to being more likely to be elevated and data from larger populations and multiple centers are needed to further verify the results. Second, some data regarding heart dysfunction, such as echocardiography, magnetic resonance, and electrocardiography, were incomplete due to the limited conditions in the isolation ward. Third, timing of cardiac markers elevation was not directly correlated with timing of the clinical worsening of the patient, i.e. patients with high BNP's on admission may already have had more severe disease and so it is simply correlative.

## Conclusions

COVID-19 patients with pre-existing CAD represented a higher abnormal percentage of cardiac markers, accompanied by high mortality and ICU admission rate. Of note, COVID-19 patients without pre-existing CAD who represented abnormal levels of cardiac markers also had high mortality. BNP together with hs-TNI, α-HBDH, CK-MB, and LDH could be a prognostic biomarker for early warning of high mortality risk through the first week.

## Supplementary Information

**Additional file 1: Table S1**. The number of patients admitted to ICU or not with abnormal cardiac markers within the first week after admission.

**Additional file 2: Figure S1**. The ICU admission rate of COVID-19 patients with cardiac markers abnormality.

**Additional file 3: Figure S2**. Median levels of the cardiac markers on the first day after admission for four groups of patients (non-survivors with CAD, survivors with CAD, non-survivors without CAD, survivors without CAD). The dotted line shows the upper reference limit of the corresponding marker.

**Additional file 4: Figure S3**. The fluctuation of serum levels for 5 cardiac markers in survivors and non-survivors with and without CAD during hospitalization. The x-axis represents the admission time. The red dotted line represents the reference limit of the marker. (Number): the fold change for median level versus the reference limit value for each marker.

**Additional file 5: Figure S4**. Risk-Stratification biomarker for COVID-19 patients for each cardiac marker. Kaplan–Meier estimates for severe/critical patients by levels of BNP, hs-TNI, α-HBDH, CK-MB, and LDH within the first week after admission in univariate Cox regression analysis.

**Additional file 6: Figure S5**. ROC curve of BNP, hs-TNI, α-HBDH, CK-MB, and LDH within the first week after admission to predict survivors and non-survivors.

**Additional file 7: Figure S6**. Correlation between five cardiac markers and CRP (a), IL-6 (b), D-dimer (c), and LYM% (d). The 95% confidence interval represented by shaded regions.

## Data Availability

The datasets used and/or analyzed during the current study are available from the corresponding author on reasonable request.

## Abbreviations

COVID-19: Coronavirus disease
CAD: Coronary artery disease
SARS-CoV-2: Severe acute respiratory syndrome coronavirus 2
hs-TNI: High-sensitivity troponin I
α-HBDH: α-Hydroxybutyrate dehydrogenase
CK-MB: Creatine kinase-MB
LDH: Lactate dehydrogenase
BNP: Brain natriuretic peptide
CT: Computed tomography
ICU: Intensive care unit
scRNA-seq: Single-cell RNA sequencing
GEO: Gene expression omnibus
ROC: Receiver operating characteristic
CRP: C-reactive protein
IL-6: Interleukin-6
PCT: Procalcitonin
NEUT%: Percentages of neutrophils
LYM%: Percentages of lymphocytes
MONO%: Percentages of monocytes
IQR: Interquartile range

## References

[1] Guan WJ, Ni ZY, Hu Y, Liang WH, Ou CQ, He JX, Liu L, Shan H, Lei CL, Hui DSC (2020). Clinical characteristics of coronavirus disease 2019 in China. N Engl J Med.

[2] Fu Y, Cheng Y, Wu Y (2020). Understanding SARS-CoV-2-mediated inflammatory responses: from mechanisms to potential therapeutic tools. Virol Sin.

[3] Singhal T (2020). A review of coronavirus disease-2019 (COVID-19). Indian J Pediatr.

[4] Zou X, Chen K, Zou J, Han P, Hao J, Han Z (2020). Single-cell RNA-seq data analysis on the receptor ACE2 expression reveals the potential risk of different human organs vulnerable to 2019-nCoV infection. Front Med.

[5] Wang D, Hu B, Hu C, Zhu F, Liu X, Zhang J, Wang B, Xiang H, Cheng Z, Xiong Y (2020). Clinical characteristics of 138 hospitalized patients with 2019 novel coronavirus-infected pneumonia in Wuhan, China. JAMA.

[6] Huang C, Wang Y, Li X, Ren L, Zhao J, Hu Y, Zhang L, Fan G, Xu J, Gu X (2020). Clinical features of patients infected with 2019 novel coronavirus in Wuhan, China. Lancet.

[7] Guo T, Fan Y, Chen M, Wu X, Zhang L, He T, Wang H, Wan J, Wang X, Lu Z (2020). Cardiovascular implications of fatal outcomes of patients with coronavirus disease 2019 (COVID-19). JAMA Cardiol.

[8] Dong N, Cai J, Zhou Y, Liu J, Li F (2020). End-stage heart failure with COVID-19: strong evidence of myocardial injury by 2019-nCoV. JACC Heart Fail.

[9] Bodor GS (2016). Biochemical markers of myocardial damage. EJIFCC.

[10] Doust JA, Pietrzak E, Dobson A, Glasziou PP (2005). How well does B-type natriuretic peptide predict death and cardiac events in patients with heart failure: systematic review. Brit. Med. J..

[11] Lee YJ, Lee J, Park YS, Lee SM, Yim JJ, Yoo CG, Kim YW, Han SK, Lee CH (2013). Predictors of cardiogenic and non-cardiogenic causes in cases with bilateral chest infiltrates. Tuberc. Respir. Dis. (Seoul).

[12] Shi S, Qin M, Shen B, Cai Y, Liu T, Yang F, Gong W, Liu X, Liang J, Zhao Q (2020). Association of cardiac injury with mortality in hospitalized patients with COVID-19 in Wuhan, China. JAMA Cardiol.

[13] Zheng Z, Peng F, Xu B, Zhao J, Liu H, Peng J, Li Q, Jiang C, Zhou Y, Liu S (2020). Risk factors of critical & mortal COVID-19 cases: a systematic literature review and meta-analysis. J Infect.

[14] Xu X, Chen P, Wang J, Feng J, Zhou H, Li X, Zhong W, Hao P (2020). Evolution of the novel coronavirus from the ongoing Wuhan outbreak and modeling of its spike protein for risk of human transmission. Sci China Life Sci.

[15] Wu F, Zhao S, Yu B, Chen YM, Wang W, Song ZG, Hu Y, Tao ZW, Tian JH, Pei YY (2020). A new coronavirus associated with human respiratory disease in China. Nature.

[16] Wrapp D, Wang NS, Corbett KS, Goldsmith JA, Hsieh CL, Abiona O, Graham BS, McLellan JS (2020). Cryo-EM structure of the 2019-nCoV spike in the prefusion conformation. Science.

[17] Zhao Y, Zhao Z, Wang Y, Zhou Y, Ma Y, Zuo W (2020). Single-cell RNA expression profiling of ACE2, the receptor of SARS-CoV-2. Am J Respir Crit Care Med.

[18] Zou X, Chen K, Zou J, Han P, Hao J, Han Z (2020). Single-cell RNA-seq data analysis on the receptor ACE2 expression reveals the potential risk of different human organs vulnerable to 2019-nCoV infection. Front Med.

[19] Li K, Huang B, Wu M, Zhong A, Li L, Cai Y, Wang Z, Wu L, Zhu M, Li J (2020). Dynamic changes in anti-SARS-CoV-2 antibodies during SARS-CoV-2 infection and recovery from COVID-19. Nat Commun.

[20] Halushka PV, Goodwin AJ, Halushka MK (2019). Opportunities for microRNAs in the crowded field of cardiovascular biomarkers. Annu Rev Pathol.

[21] Kang H (2013). The prevention and handling of the missing data. Korean J Anesthesiol.

[22] Gauthier J, Wu QV, Gooley TA (2020). Cubic splines to model relationships between continuous variables and outcomes: a guide for clinicians. Bone Marrow Transpl.

[23] Wu S, Du Z, Shen S, Zhang B, Yang H, Li X, Cui W, Cheng F, Huang J (2020). Identification and validation of a novel clinical signature to predict the prognosis in confirmed coronavirus disease 2019 patients. Clin Infect Dis.

[24] Li ZS, Li XY, Zhang XQ, Chen P, Wang B, Chen XF, Han H, Zhou FJ (2020). Prognostic significance of common preoperative laboratory variables in penile squamous cell carcinoma. Int J Urol.

[25] Wang L, Yu P, Zhou B, Song J, Li Z, Zhang M, Guo G, Wang Y, Chen X, Han L (2020). Single-cell reconstruction of the adult human heart during heart failure and recovery reveals the cellular landscape underlying cardiac function. Nat Cell Biol.

[26] Butler A, Hoffman P, Smibert P, Papalexi E, Satija R (2018). Integrating single-cell transcriptomic data across different conditions, technologies, and species. Nat Biotechnol.

[27] Li L, Wang L, Li H, Han X, Chen S, Yang B, Hu Z, Zhu H, Cai C, Chen J (2018). Characterization of LncRNA expression profile and identification of novel LncRNA biomarkers to diagnose coronary artery disease. Atherosclerosis.

[28] South AM, Diz DI, Chappell MC (2020). COVID-19, ACE2, and the cardiovascular consequences. Am J Physiol Heart Circ Physiol.

[29] Zhang H, Penninger JM, Li Y, Zhong N, Slutsky AS (2020). Angiotensin-converting enzyme 2 (ACE2) as a SARS-CoV-2 receptor: molecular mechanisms and potential therapeutic target. Intensive Care Med.

[30] Stopsack KH, Mucci LA, Antonarakis ES, Nelson PS, Kantoff PW (2020). TMPRSS2 and COVID-19: serendipity or opportunity for intervention?. Cancer Discov.

[31] Kawase M, Shirato K, van der Hoek L, Taguchi F, Matsuyama S (2012). Simultaneous treatment of human bronchial epithelial cells with serine and cysteine protease inhibitors prevents severe acute respiratory syndrome coronavirus entry. J Virol.

[32] Zhou Y, Vedantham P, Lu K, Agudelo J, Carrion R, Nunneley JW, Barnard D, Pohlmann S, McKerrow JH, Renslo AR (2015). Protease inhibitors targeting coronavirus and filovirus entry. Antiviral Res.

[33] Dong Y, Li X, Yu Y, Lv F, Chen Y (2020). JAK/STAT signaling is involved in IL-35-induced inhibition of hepatitis B virus antigen-specific cytotoxic T cell exhaustion in chronic hepatitis B. Life Sci.

[34] Lippi G, Lavie CJ, Sanchis-Gomar F (2020). Cardiac troponin I in patients with coronavirus disease 2019 (COVID-19): evidence from a meta-analysis. Prog Cardiovasc Dis.

[35] Bao C, Tao X, Cui W, Yi B, Pan T, Young KH, Qian W (2020). SARS-CoV-2 induced thrombocytopenia as an important biomarker significantly correlated with abnormal coagulation function, increased intravascular blood clot risk and mortality in COVID-19 patients. Exp Hematol Oncol.

