# Supplementary material for: Implications of cardiac markers in risk-stratification and management for COVID-19 patients
Source: Crit Care. 2021 Apr 26;25:158. doi: 10.1186/s13054-021-03555-z (PMC8074282; doi:10.1186/s13054-021-03555-z)
Supplement: Supplementary file 1 — Additional file 1: Table S1. The number of patients admitted to ICU or not with abnormal cardiac markers within the first week after admission. [file 13054_2021_3555_MOESM1_ESM.docx]

**Table S1. The number of patients admitted to ICU or not with abnormal cardiac markers within the first week after admission.**

| **Cardiac marker** | **In ICU** | **Not in ICU** | **Total** |
| --- | --- | --- | --- |
| BNP | 35 (28.00%) | 90 (72.00%) | 125 |
| hs-TNI | 20 (38.46%) | 32 (61.54%) | 52 |
| α-HBDH | 88 (18.72%) | 382 (81.28%) | 470 |
| CK-MB | 31 (39.74%) | 47 (60.26%) | 78 |
| LDH | 80 (20.78%) | 305 (79.22%) | 385 |
