# Supplementary figures and images for: Implications of cardiac markers in risk-stratification and management for COVID-19 patients
Source: Crit Care. 2021 Apr 26;25:158. doi: 10.1186/s13054-021-03555-z (PMC8074282; doi:10.1186/s13054-021-03555-z)

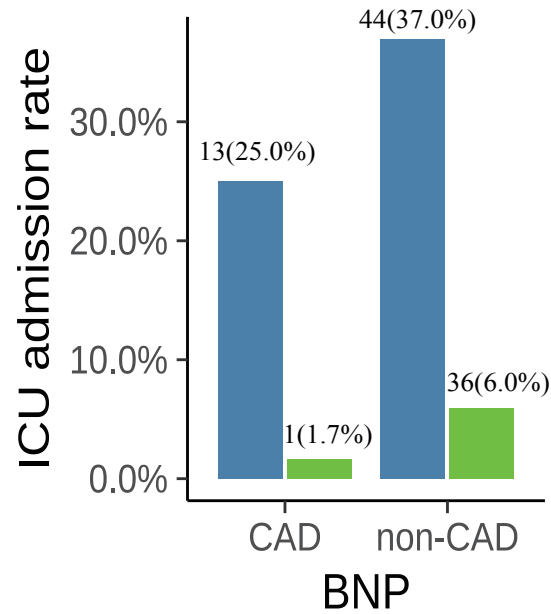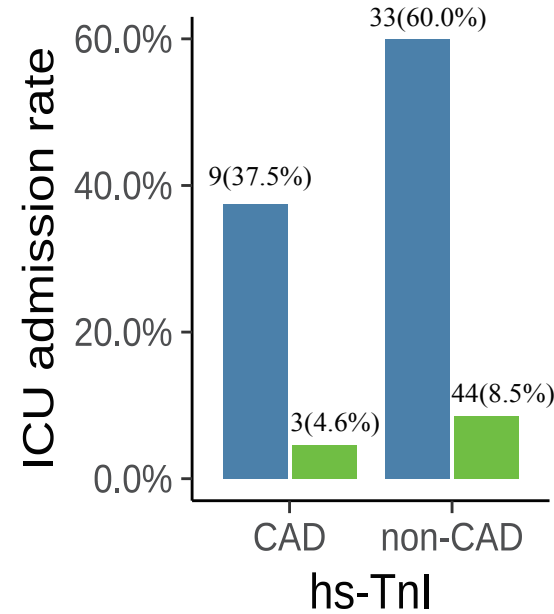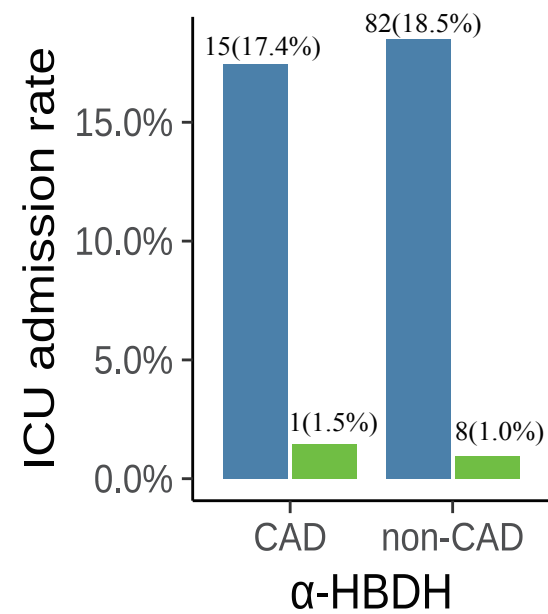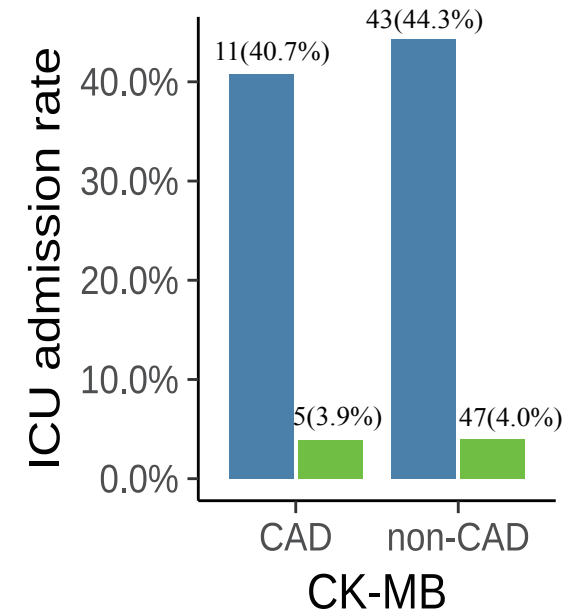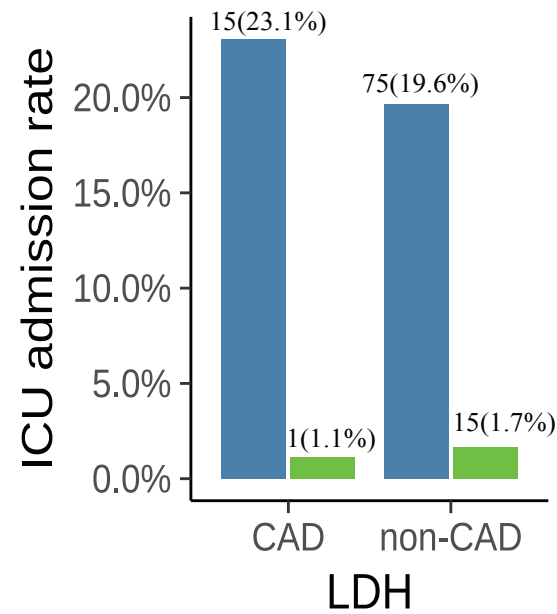

Abnormal Normal

Supplement: Supplementary file 2 — Additional file 2: Figure S1. The ICU admission rate of COVID-19 patients with cardiac markers abnormality. [file 13054_2021_3555_MOESM2_ESM.pdf]

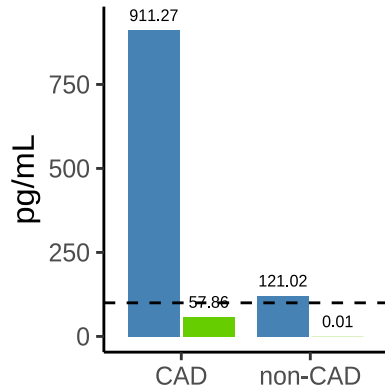

BNP

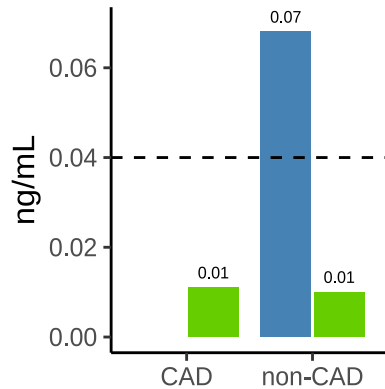

hs-TnI

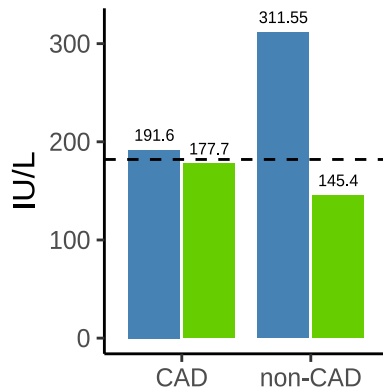

α-HBDH

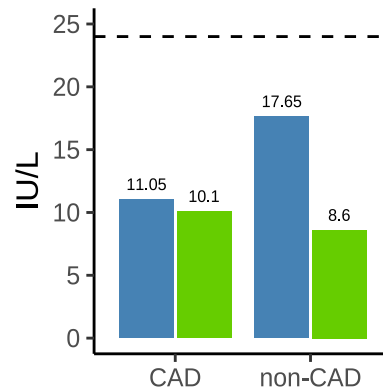

CK-MB

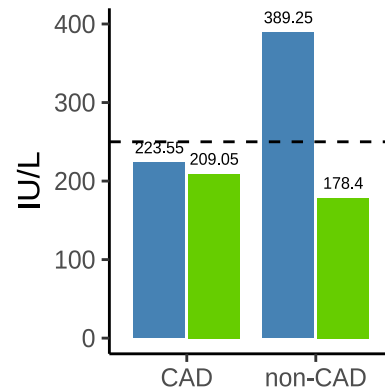

LDH

■ Non-survivors ■ Survivors

Supplement: Supplementary file 3 — Additional file 3: Figure S2. Median levels of the cardiac markers on the first day after admission for four groups of patients (non-survivors with CAD, survivors with CAD, non-survivors without CAD, survivors without CAD). The dotted line shows the upper reference limit of the corresponding marker. [file 13054_2021_3555_MOESM3_ESM.pdf]

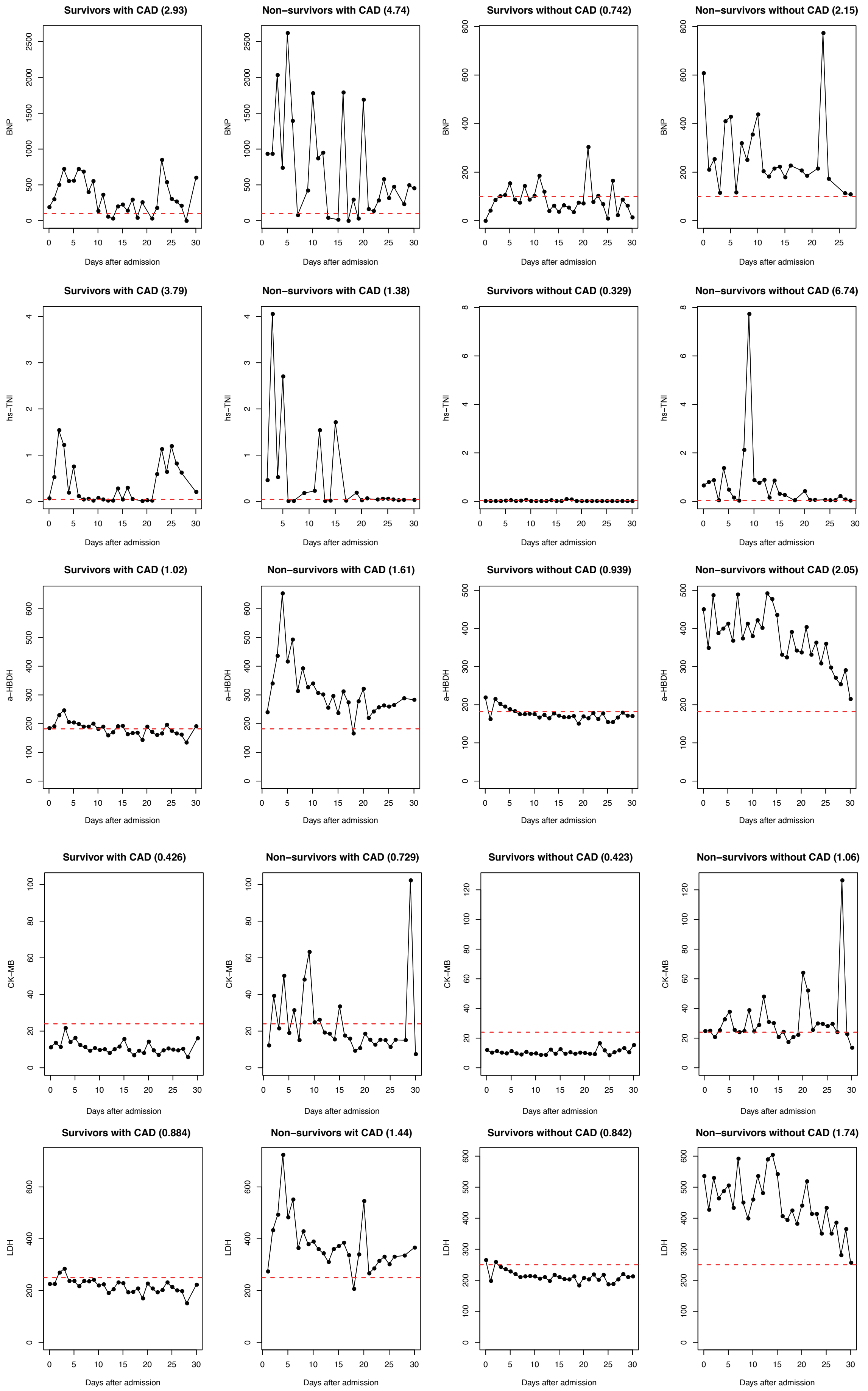

Supplement: Supplementary file 4 — Additional file 4: Figure S3. The fluctuation of serum levels for 5 cardiac markers in survivors and non-survivors with and without CAD during hospitalization. The x-axis represents the admission time. The red dotted line represents the reference limit of the marker. (Number): the fold change for median level versus the reference limit value for each marker. [file 13054_2021_3555_MOESM4_ESM.pdf]

**BNP**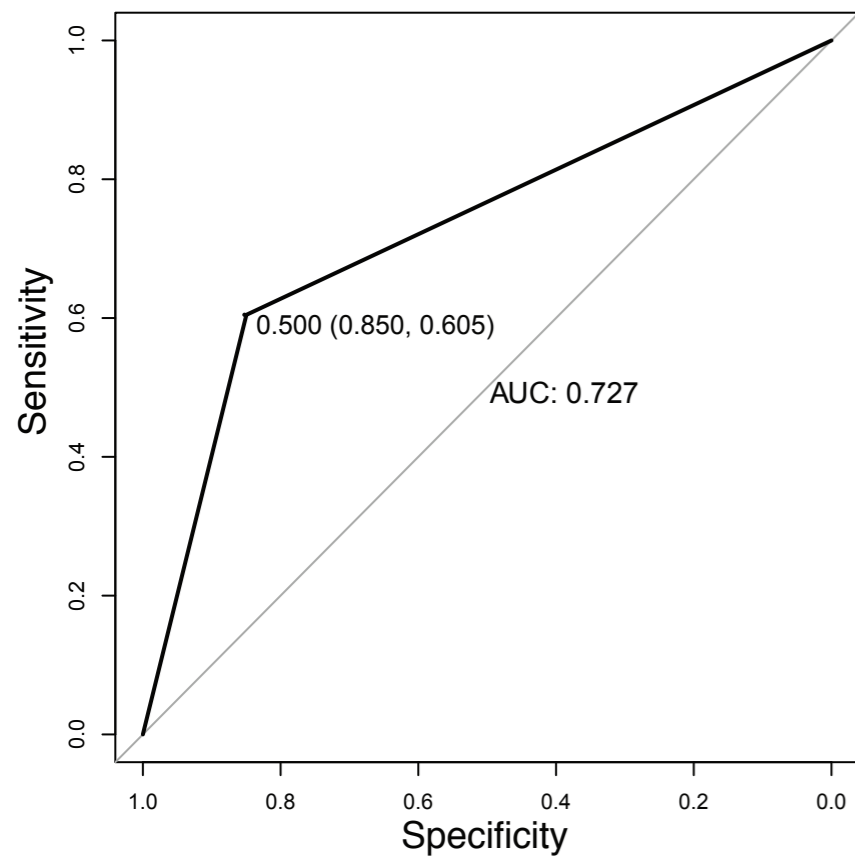**hs-TNI**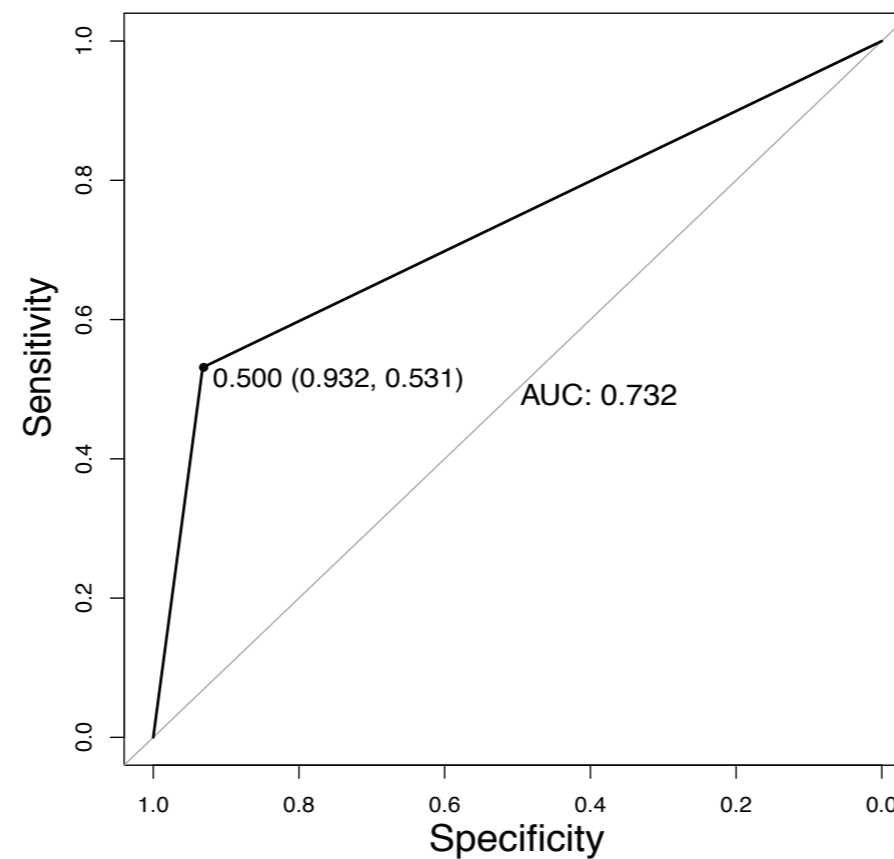 **$\alpha$ -HBDH**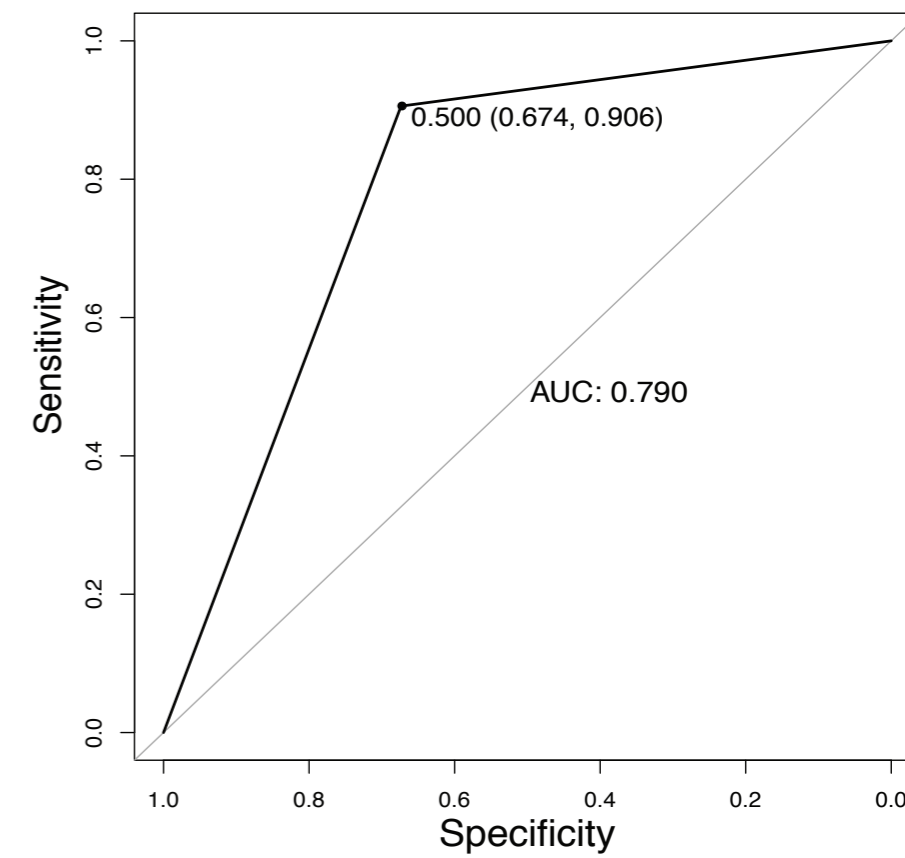**CK-MB**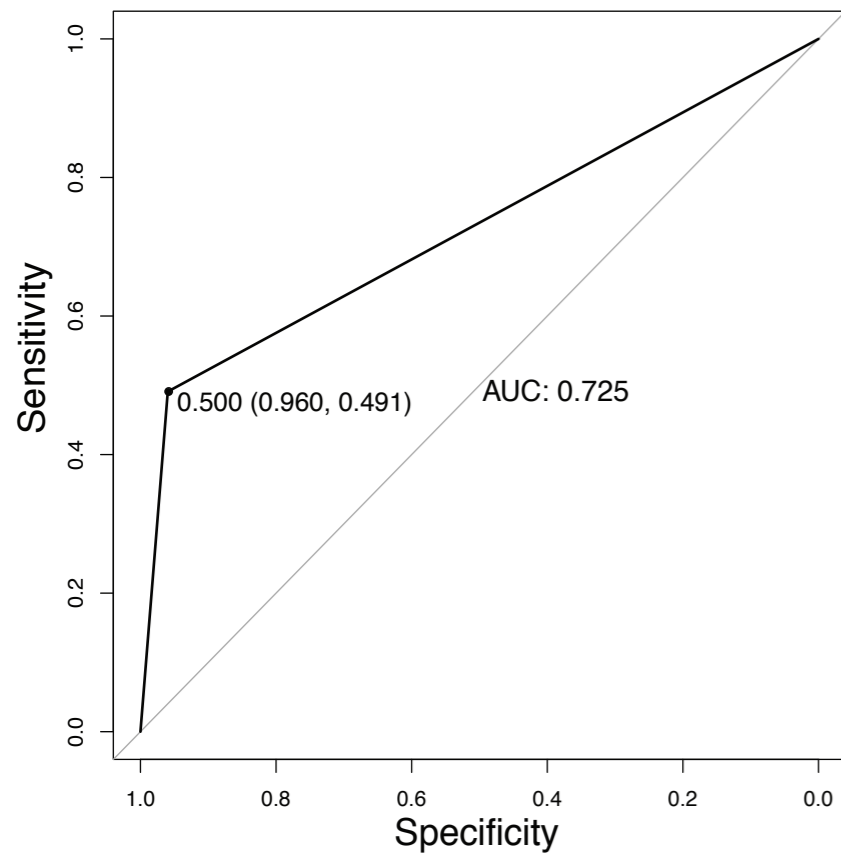**LDH**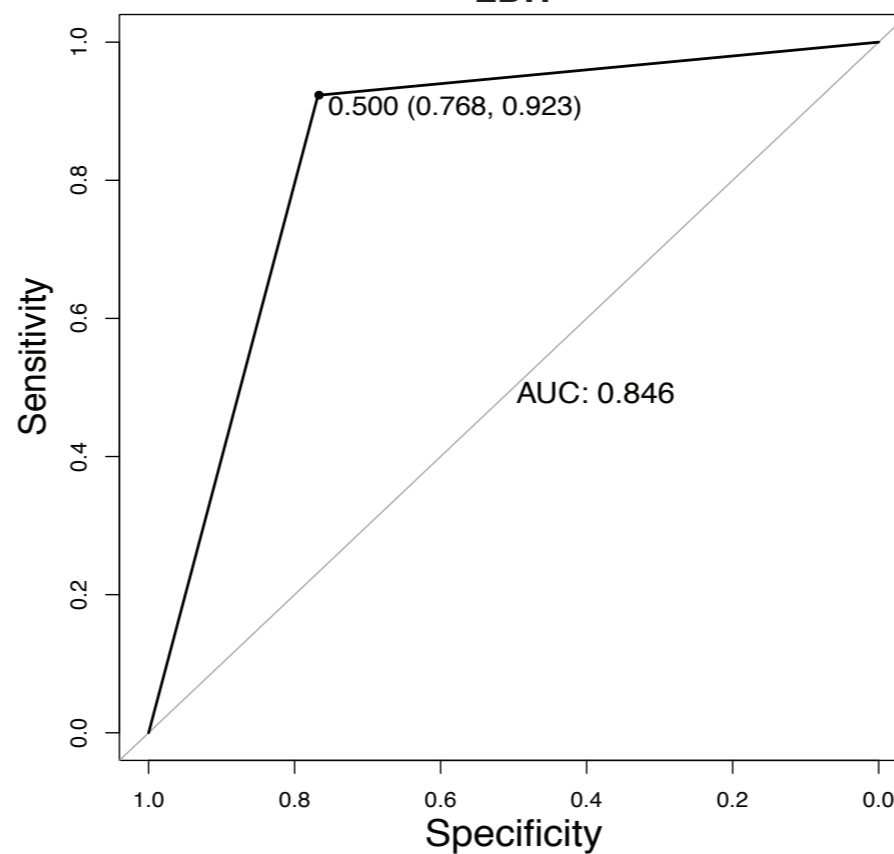

Supplement: Supplementary file 6 — Additional file 6: Figure S5. ROC curve of BNP, hs-TNI, α-HBDH, CK-MB, and LDH within the first week after admission to predict survivors and non-survivors. [file 13054_2021_3555_MOESM6_ESM.pdf]

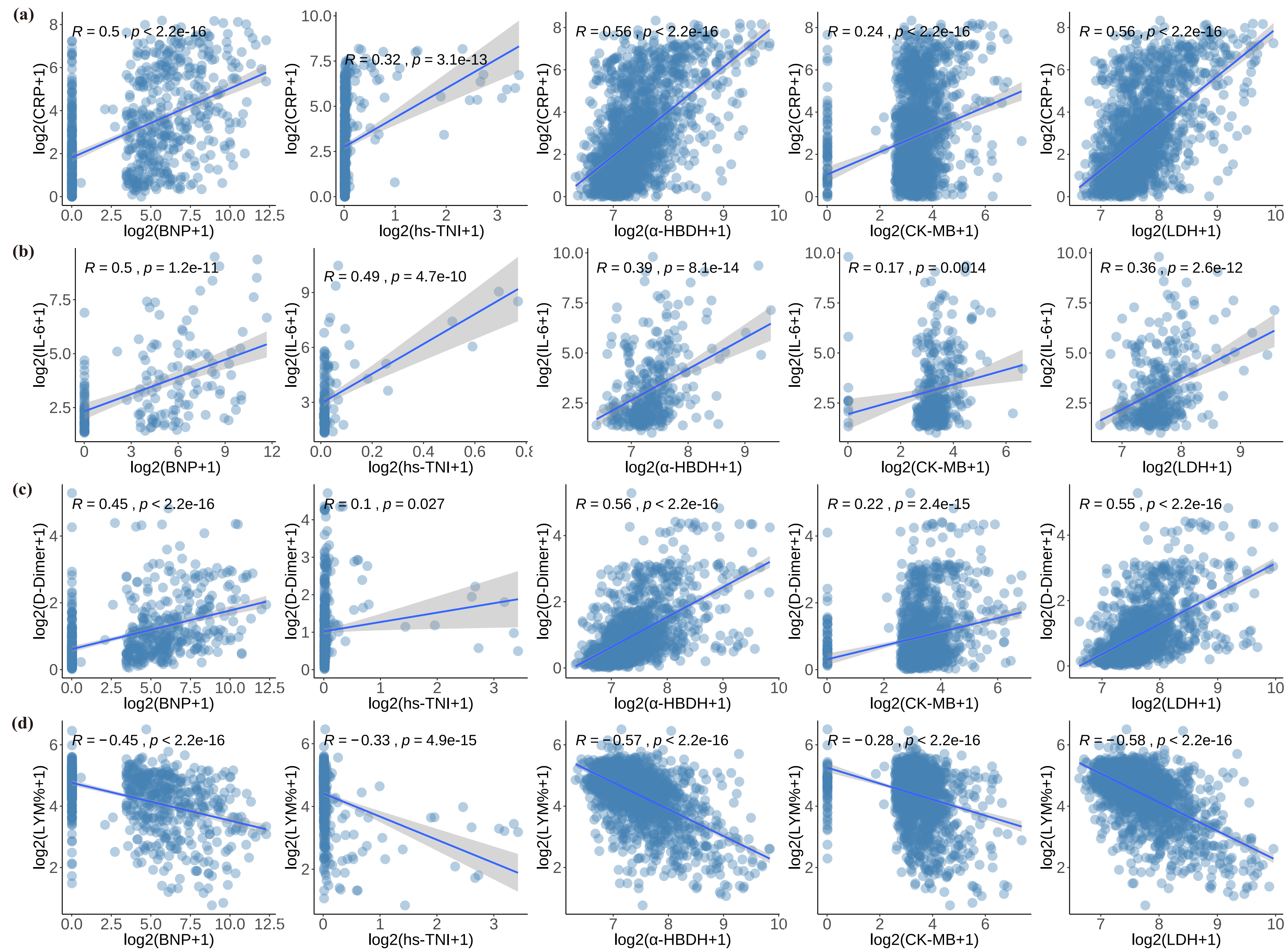

Supplement: Supplementary file 7 — Additional file 7: Figure S6. Correlation between five cardiac markers and CRP (a), IL-6 (b), D-dimer (c), and LYM% (d). The 95% confidence interval represented by shaded regions. [file 13054_2021_3555_MOESM7_ESM.pdf]
