# Supplementary material for: Implications of cardiac markers in risk-stratification and management for COVID-19 patients
Source: Crit Care. 2021 Apr 26;25:158. doi: 10.1186/s13054-021-03555-z (PMC8074282; doi:10.1186/s13054-021-03555-z)
Supplement: Supplementary file 5 — Additional file 5: Figure S4. Risk-Stratification biomarker for COVID-19 patients for each cardiac marker. Kaplan–Meier estimates for severe/critical patients by levels of BNP, hs-TNI, α-HBDH, CK-MB, and LDH within the first week after admission in univariate Cox regression analysis. [file 13054_2021_3555_MOESM5_ESM.pdf]

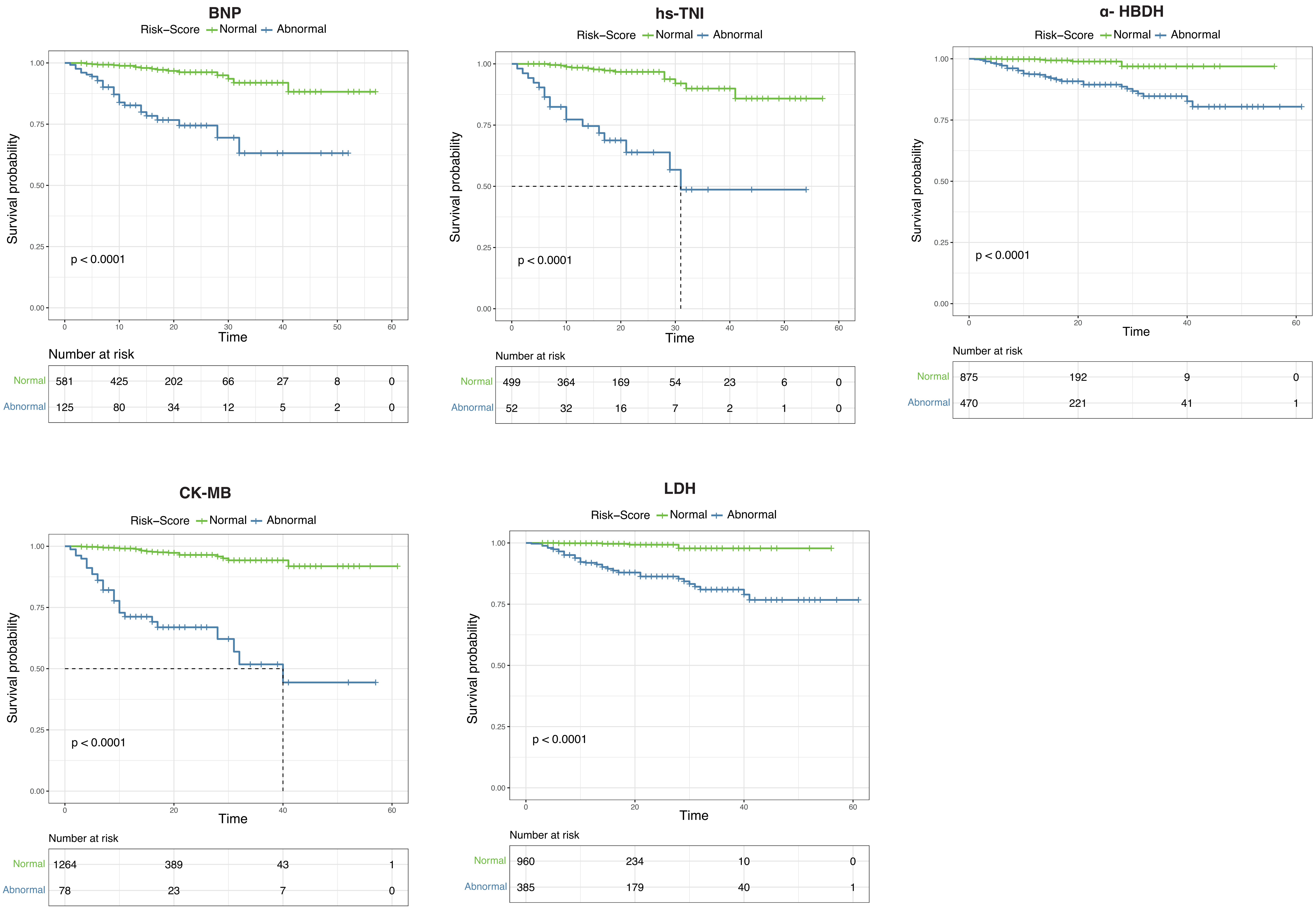
BNP
Risk−Score Normal Abnormal
Survival probability
p < 0.0001
Time
Number at risk
Normal 581 425 202 66 27 8 0
Abnormal 125 80 34 12 5 2 0
hs-TNI
Risk−Score Normal Abnormal
Survival probability
p < 0.0001
Time
Number at risk
Normal 499 364 169 54 23 6 0
Abnormal 52 32 16 7 2 1 0
α- HBDH
Risk−Score Normal Abnormal
Survival probability
p < 0.0001
Time
Number at risk
Normal 875 192 9 0
Abnormal 470 221 41 1
CK-MB
Risk−Score Normal Abnormal
Survival probability
p < 0.0001
Time
Number at risk
Normal 1264 389 43 1
Abnormal 78 23 7 0
LDH
Risk−Score Normal Abnormal
Survival probability
p < 0.0001
Time
Number at risk
Normal 960 234 10 0
Abnormal 385 179 40 1
